# MRI methods for the study of central nervous system involvement in autonomic function

**DOI:** 10.3389/fnetp.2026.1812968

**Published:** 2026-05-07

**Authors:** Jeff Duyn

**Affiliations:** Advanced MRI Section, Laboratory of Functional and Molecular Imaging, National Institute of Neurological Disorders and Stroke, National Institutes of Health, Bethesda, MD, United States

**Keywords:** autonomic physiology, autonomic regulation, brain function, central autonomic network, CNS, MRI, network physiology

## Abstract

To support healthy bodily functioning over a great variety of physiological and psychological conditions, mammalian autonomic regulation (AR) relies on a highly complex and incompletely explored nervous network that includes components of central and peripheral nervous systems. While, in animal, many components of this network and their interactions have been mapped with invasive electrophysiology and optical studies as well as with staining and tracing techniques, these findings only partly transfer the human. With the advent of non-invasive neuroimaging approaches based on MRI, much is being learned about AR in human, including the extent of CNS involvement, and the potential relevance of AR for memory function and brain waste clearance. In this review, some of these developments are highlighted, with an emphasis on methodological aspects.

## 1 Introduction

Autonomic regulation (AR) serves to maintain physiological homeostasis of the body’s organs. It relies, in part, on a complex network of specialized regions in the central nervous system (CNS) often indicated as the central autonomic network (CAN) (Benarroch, 1993; Valenza et al., 2025). This network consists of multiple, highly interconnected forebrain, midbrain and brainstem regions that together process afferent viscero-sensory and humoral signals to adjust activity of effector organs when conditions demand. While AR has been extensively studied in animal with invasive electrophysiology and optical studies, as well as with staining and tracing techniques (see, e.g., (Dampney and Horiuchi, 2003; Guyenet et al., 1989; Verberne and Owens, 1998)), detailed understanding of AR in human has lagged. In part, this is because animal findings do not generally translate to human, and limited opportunity exists for invasive studies that would be needed to comprehensively study the joint humoral, metabolic, electrical, and chemical activity changes in the CNS and its input (sensor) and output (effector) organs.

Nevertheless, much has been learned over the last few decades from MRI studies, supplementing information from animal research as well as lesion and pathologic studies in human. MRI studies have allowed study of brain physiology and anatomy from an increasing number of aspects and in ever increasing detail. In large part, these approaches use anatomical MRI, diffusion weighted MRI (DW MRI), and functional MRI (fMRI) to study the extent and connectivity of the brain network involved with autonomic regulation. After briefly reviewing these MRI approaches and their main findings, I will discuss some recent fMRI findings on the association between autonomic activity, arousal state and memory function. Briefly discussed here is also the autonomic involvement in brain fluid homeostasis, with potential relevance to brain waste clearance.

## 2 Overview of the CAN

Apart from maintaining homeostatic balance under stationary conditions, a major role of AR is to adjust the physiological parameters of the bodies organs to maintain proper functioning under varying demands, including the response to environmental and mental challenges and anticipated need (Theriault et al., 2025). In part, this included the generation of conscious (voluntary) or subconscious (reflex) changes in autonomic activity (Benarroch, 1993).

**FIGURE 1:**
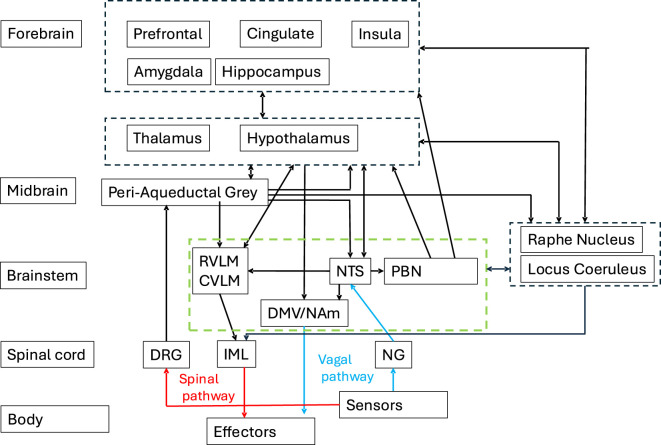
Overview of nervous system nodes involved with autonomic regulation. This highly simplified diagram only shows major nodes and connections. Dorsal root ganglion (DRG) and nodose ganglion (NG) of the PNS connect peripheral sensors to the CNS through spinal (red) and vagal (blue) pathways respectively. Similarly, the immediolateral column of the spinal cord and the dorsal motor nucleus of the vagus nerve (DMV) and the nucleus ambiguous (NAm) of the brain stem connect CNS to effector organs. RVLM = rostral ventrolateral medulla; CVLM = caudal ventrolateral medulla; NTS = nucleus of the solitary tract; PBN = parabrachial nucleus; IML = intermediolateral nucleus. Dashed boxes indicate grouping of highly interconnected nodes. Green box indicates baroreflex arc.

Effectuation of AR by the autonomic nervous system (ANS) relies on information about the body’s physiological state relayed by central and peripheral sensors. These include pressure and stretch receptors, CO_2_ and O_2_ sensors, and reporters on immune and endocrine activity (Ma et al., 2025; Engelen et al., 2023; Valenza et al., 2025). From this information, command signals are distilled to elicit autonomic adjustments through effectors such as heart, lungs and vasculature. Sensor and effector information is relayed to and from the CNS by sympathetic and parasympathetic branches of the PNS as well as by a parallel spinal pathway (Valenza et al., 2025) (Figure 1). Physiological information is also conveyed from sensors within the CNS itself that monitor, e.g., blood-borne agents (O_2_, CO_2_, neurochemicals) and pressure (Golanov and Reis, 1996; Guyenet and Stornetta, 2022).

As shown in the highly simplified schematic of Figure 1, CNS involvement in AR includes many distinct regions spread across forebrain, midbrain, brainstem, and spinal cord, many of which have been mapped by MRI. As these regions have been extensively reviewed in previous work (Benarroch, 1993; Benarroch, 2018; Dampney, 2015; Dampney, 2016; Saper, 2002), only a few aspects will be briefly highlighted here.

Notable from Figure 1 is the high connectivity between individual regions that control autonomic function, reflecting an adaptability to generate flexible autonomic adjustments, fine-tuned to the specific sensor input and fore/midbrain activity. This adaptability is achieved by differential engagement of the various regions with each type of autonomic response. One example is a differential response to psychological (pain, fear, anxiety, etc.) versus physiological stressors (Dampney, 2015), with psychological stressors involving a more elaborate and possibility initiating forebrain response including prefrontal cortex, cingulate, and insula. Responses to mild physiological stressors on the other hand would be more restricted to brainstem and midbrain regions possibly including subcortical forebrain regions (Dampney, 2015; Dampney, 2016; Blessing, 2003).

A core set of brainstem regions, including RVLM, CVLM, NTS, PBN, and NAm (Figure 1) form the so-called “baroreflex arc”, which adjusts cardio-vascular parameters to regulate blood pressure. Its sensitivity and pressure set point is set by modulatory input from locus coeruleus, raphe nucleus, PBN, hypothalamus and pedunculopontine nucleus (not shown in Figure 1) in an arousal state dependent manner (de Zambotti et al., 2018), and indirectly from forebrain regions that may include the medial prefrontal cortex and the insular cortex (Verberne and Owens, 1998). Importantly, many of these regions are also part of the arousal regulating ascending reticular activating system (RAS) (Silvani et al., 2015), which may not be surprising as several beneficial and survival-relevant behaviors require joint autonomic and cortical arousal (with the latter defined as Electroencephalography (EEG) desynchronization (Halasz et al., 2004; Silvani et al., 2015)). One example is the fight-or-flight or orienting response to fear-inducing or painful stimuli, eliciting joint changes in cortical and autonomic arousal (Cannon, 1929; Hilton, 1982). RAS and CAN are highly interconnected, with the RAS influencing the CAN’s ability to regulate the body’s internal state in response to arousal and wakefulness. Thus, autonomic arousal and cortical arousal frequently co-occur, with the extent to and the order in which regions of RAS and CAN co-activate in part dependend on the strength an type of the initiating source and brain state (de Zambotti et al., 2018).

## 3 (f)MRI approaches to study CNS involvement in AR

The ANS and its CNS components have been extensively studied in animals with a range of invasive methods such as intracortical electrophysiology, optical imaging, and intracortical flow, pressure, and chemical probes. Because of their invasiveness, extension to human studies has been limited. In this regard, the non-invasiveness of MRI relieves some of this limitation. A major strength of MRI compared to other neuroimaging methods is the flexibility by which contrast can be manipulated with the use of so-called “pulse sequences”. Popular contrasts include various types of tissue physiology (cerebral blood flow, volume, and oxygenation, water diffusion, metabolism) and anatomical structure (vasculature, microstructural properties, myelination, grey and white matter distribution). Furthermore, rapid MRI methods such as functional MRI (fMRI) allow the indirect study of dynamic changes in neural activity by measuring associated changes in blood volume, oxygenation, and flow (for review see (Glover, 2011)). Of these, blood oxygen level dependent (BOLD) contrast has been the most popular in fMRI studies of the CAN. Rapid MRI also allows the study of tissue and cerebrospinal fluid (CSF) movement associated with (neuro-)physiological processes, providing a window into possible CAN involvement with the removal of waste products from the parenchyma (Hladky and Barrand, 2022; Kipnis et al., 2025).

MRI provides excellent spatial resolution that is somewhat dependent on the type of contrast studied, the MRI field strength employed, and the strength of the magnetic field gradients that are used for image generation and contrast sensitization. High field (7 T) scanners provide around 0.4 mm resolution for most anatomical contrasts within typical scan times of a few minutes. fMRI spatial resolution is somewhat courser and typically around 1–2 mm, while temporal resolution is limited to a few (3-4) seconds. The latter is dictated not only by MRI scan speed, but also by the relative sluggishness of the hemodynamic response (HDR). The improved resolution of modern high field MRI scanners is proving beneficial for study of AR (Chang et al., 2016; Sclocco et al., 2018). New developments in gradient hardware allow further resolution improvements (Huang et al., 2021) and are expected to further facilitate study of AR.

**FIGURE 2:**
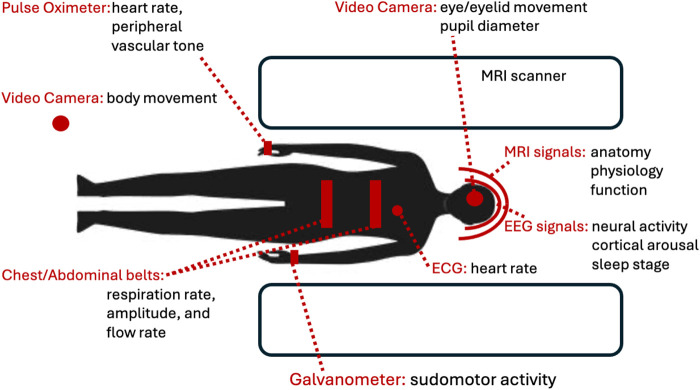
Overview of signals relevant to study of CAN with MRI. EEG and fMRI allow the study of spontaneous or task evoked brain activity from its effect on scalp electrical neural activity and brain blood flow respectively. Proper interpretation of these signals requires various physiological measurements from peripheral devices. ECG (electrocardiography) is typically part of EEG equipment and required for removal of cardioballistic artifacts from the EEG that are particularly pronounced when acquired in the MRI.

fMRI Can be used to study both spontaneously varying and task-evoked activity. Combined with measures of peripheral physiology such as respiratory flow (from, e.g., chest belt), heart rate (from electrocardiogram or photoplethysmography), skin blood volume (from photoplethysmography), blood pressure (from photoplethysmography, others), sudomotor activity (from skin electrical conductivity), and pupil diameter (from eye camera), fMRI provides a great opportunity to study CNS involvement in AR (Figure 2).

Although the fMRI signal can be used to map neural activity in the CAN, it is also sensitive to vascular changes elicited by effector organs controlled by the CAN. For example, blood pressure changes due to cardiac rate or ejection fraction changes may by themselves affect the fMRI signal, which therefore cannot directly be interpreted as reflecting local electrocortical activity. Similarly, intravascular CO_2_ changes with changes in respiration may affect fMRI through changes in vascular tone, hence blood flow and oxygenation (Birn et al., 2006). Sympathetic vasoconstriction may also affect fMRI independent of local neuronal activity (Ozbay et al., 2019). Lastly, during, e.g., arousal fluctuations (see below), changes in CAN activity may be accompanied by activity in cortical arousal-related regions, confounding the mapping of CAN. Thus, caution should be exercised with interpreting fMRI as various neuronal and non-neuronal sources may contribute (Duyn, 2011).

### 3.1 Anatomy and structural connectivity of autonomic brainstem nuclei (SWI/DTI)

A major aspect of the study of the CAN with MRI is identifying the location of its various constituent nodes and their structural connectivity. This information can subsequently be used to interpret fMRI studies of activity changes during elicited or spontaneous changes in autonomic function.

Many of the major forebrain nodes of the CAN can be readily identified from conventional MRI scans of modest spatial resolution and with good grey-white matter contrast based on sensitization to T_1_ relaxation time differences (Mugler and Brookeman, 1990). Visualization of the many brainstem nuclei of the CAN however is more challenging due to their small size and modest T_1_ contrast. To overcome this, high resolution (<0.5 mm) magnetic susceptibility weighting can be used, a contrast that is sensitive to tissue variations in paramagnetic species such as venous blood and iron (Haacke et al., 2004). Recent high field susceptibility weighted MRI studies on 7.0 T scanners have been particularly successful in this regard, helped by increases in both intrinsic MRI sensitivity and contrast with increasing MRI field strength. This has allowed the identification of many of the CAN related nuclei based on comparison with knowledge from *post mortem* studies (Singh et al., 2021; Garcia-Gomar et al., 2019).

In addition to map nodes of the CAN, MRI can also be used to investigate the major structural connections between them. DW MRI can be used to study white matter fiber orientation based on a relatively strong diffusion of water along fiber axes, information that can be exploited to track (mostly myelinated) fibers coursing through the brain (Conturo et al., 1999; Mori et al., 1999). Structural connectivity of the CAN can then be analyzed either examining these fibers between various nodes selected from anatomical MRI, or alternatively aided by functional MRI (Goffi et al., 2024). Results from such studies (Singh et al., 2022; Garcia-Gomar et al., 2022; Edlow et al., 2016) have revealed a high connectivity within and beyond the brainstem, confirming the large existing literature on tracing and staining studies in non-human primates. Like anatomical studies based on T_1_ and magnetic susceptibility contrast, structural connectivity studies with DW MRI of the brainstem are challenging due to the small size of autonomic nuclei and their (many) interconnecting fiber bundles.

### 3.2 Functional studies (fMRI)

Human studies of the CAN with fMRI have been used to map central involvement with various aspects of autonomic activity, either elicited by tasks, or occurring spontaneously. A primary goal of this work has been to confirm and refine knowledge derived from animal studies, and to develop a better understanding of CAN function in health and disease. These studies provide a general, course overview of brain regions involved with autonomic function in health and pathologies such as Alzheimer’s disease and epilepsy. In general, they rely on correlating autonomic measures derived from peripheral physiology with the fMRI signal, either during rest or tasks designed to elicit an autonomic response. In the following these approaches will be discussed separately.

#### 3.2.1 fMRI of CAN/autonomic changes elicited by tasks

A large literature reports on studies that have used fMRI to map CAN activity, with the earliest studies focusing on the brainstem (Harper et al., 2000; Topolovec et al., 2004; McKay et al., 2003). A large majority has focused on CAN activity elicited by tasks, and these have been comprehensively reviewed previously (Sklerov et al., 2019; Shoemaker and Goswami, 2015; Kimmerly, 2017; Gianaros and Sheu, 2009; Park and Thayer, 2014). A great variety of tasks/stimuli have been used to elicit CAN activity, including those that target specific afferent nerves (e.g., vagus-, trigeminal-, and occipital nerves) (Frangos et al., 2015; Borgmann et al., 2021; Kraus et al., 2013; Mehnert et al., 2024), visceral stimuli (Topolovec et al., 2004), blood pressure challenges (Harper et al., 2000), respiratory challenges (McKay et al., 2003), static handgrip exercise (Sander et al., 2010), pain (Sclocco et al., 2016), and psychological stress (Erhart et al., 2024; Gianaros and Sheu, 2009).

A main takeaway from fMRI studies of the CAN they to large extent replicate invasive studies of non-human primates and rodents, covering all areas indicated in Figure 1. Meta-analysis of task studies suggests that involvement in human may extend to other areas as well, including angular gyrus, pulvinar, and precuneus (Beissner et al., 2013). Importantly, observed involvement appears dependent on what neural process are generated by the task in addition to those strictly involved with AR with important differences seen between the response to physiological versus psychological stimuli (Cechetto and Shoemaker, 2009). These differences may in part relate to the relative contribution of sympathetic versus parasympathetic activity to AR (Beissner et al., 2013).

A remaining difficulty with the interpretation of fMRI of the CAN relates to spatial and temporal resolution constraints. For example, neural circuitry within each of the nodes shown in Figure 1 is highly complex, often combining excitatory and inhibitory components and multiple neurotransmitter types over sub-millimeter spatial scales. This detail is typically lost at even highest fMRI spatial resolutions available at high field. In addition, inference of any causal interaction between network nodes from temporal sequence in their activity is complicated by their high interconnectivity and the poor, seconds-scale temporal resolution of the hemodynamics underlying fMRI contrast. Detailed discussion of these limitations can be found in prior work (Cauzzo et al., 2022; Sclocco et al., 2018).

#### 3.2.2 fMRI of CAN/autonomic variability during awake rest

In addition to allowing the study of CAN based on evoked autonomic activity, fMRI can also be used to study CAN from spontaneous autonomic variability (Macefield and Henderson, 2010; James et al., 2013). A potential advantage of resting state studies is that they facilitate the study of the dependence of CAN function on arousal state, e.g., across the sleep-wake cycle (see below). As with task-evoked studies, task-free or “resting state” studies map CAN involvement in AR by comparing autonomic indicators such as heart rate and its variability, respiratory flow rate, blood pressure, and muscle sympathetic nerve activity with the fMRI signal (Napadow et al., 2008; Sklerov et al., 2019; Matusik et al., 2023). This can be done by, for example, temporally correlating these signals or using these signals as regressors in a generalized linear model. Alternatively, one can study how the correlation between fMRI signals themselves (also called “functional connectivity”) depends on autonomic indicators. An example is the study of sympathetic versus parasympathetic CAN contributions to AR based on the analysis of heart rate variability, with variability at the respiratory rate indicative of a parasympathetic dominance (Chang et al., 2013; Duggento et al., 2016; Valenza et al., 2024).

Like task-evoked studies, resting state studies have confirmed the involvement of many brain regions in AR, including those shown in Figure 1 (Sklerov et al., 2019). It is important to consider however that interpretation of fMRI studies of CAN, especially during the resting state should be done with caution, as there are a number of potential confounds. First, fMRI activity that correlates with autonomic measures does not necessarily reflect activity in regions strictly involved with AR: as mentioned above, other processes (e.g., psychological activity or cortical arousal) may co-fluctuate with autonomic activity (Bolt et al., 2025). This will be discussed in more detail below. Next, fMRI does not resolve how a region’s activity contributes to AR: for example, activity in insular cortex or PAG may support both sensing and effector signaling function (Faull et al., 2015). Similarly, fMRI activity may reflect inhibitory versus excitatory function or support sympathetic versus parasympathetic activity: these latter two often have antagonistic effects on AR. Lastly, as briefly indicated above and detailed in the next section, there are non-neuronal contributions to the fMRI signal that depend on autonomic activity and therefore can confound correlation analysis.

### 3.3 CAN activity and arousal state changes

As indicated above, the precise involvement of CAN’s various nodes in AR is context dependent, for example, the specific autonomic challenge or demand and the arousal state of the brain. Furthermore, changes in autonomic activity often are accompanied by changes in cortical arousal (and *vice versa*) due to overlapping and highly interacting neural substrates that elicit these changes (Silvani et al., 2015; Scammell et al., 2017) including the above mentioned overlap of baroreflex arc and RAS (Benarroch, 2018). Functionally, the RAS provides input to the CAN through its neuromodulatory projections to the hypothalamus (and through raphe nucleus and locus coeruleus, see Figure 1) and other subcortical structures (Foote and Morrison, 1987; Pourmotabbed et al., 2025). In addition, there is extensive interconnectivity between RAS and CAN through forebrain regions.

**FIGURE 3:**
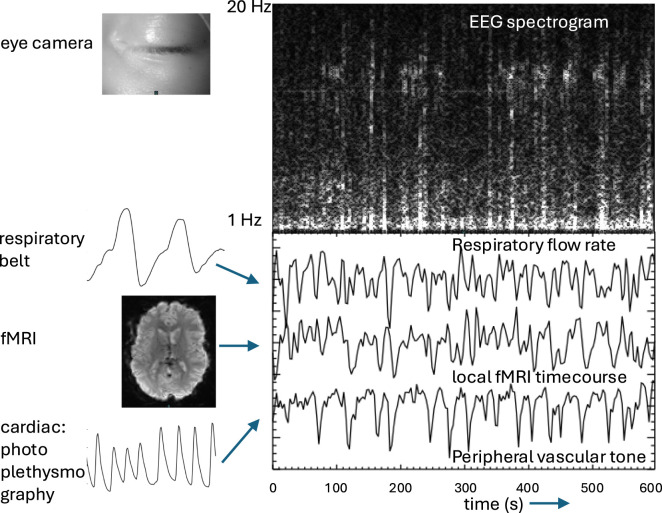
Example of some of the signals collected for study of CAN with fMRI. EEG spectrogram shows a 10-min segment of light sleep with intermittent spindle and slow wave activity. Concurrently acquired fMRI, respiratory belt signals, and photoplethysmography are analyzed to interpret local fMRI signal changes and their association with autonomic activity reflected in changes in respiratory flow rate and peripheral vascular tone.

Due to this tight integration between CAN and RAS, autonomic changes are often accompanied by widespread forebrain activity changes involvement of fMRI signal with arousal changes. This complicates the interpretation of fMRI-based mapping of the CAN, as its activity can be confounded by modulatory changes in cortical activity. In addition, as mentioned above, autonomic activity can also affect the fMRI signal in a widespread manner through non-neuronal pathways (Chang et al., 2016; Bolt et al., 2025). For example, intravascular CO_2_ changes with changes in respiration may affect fMRI in a widespread manner due to vasodilatory effects on cerebral blood flow and oxygenation (Birn et al., 2008). Sympathetic vasoconstriction may also affect fMRI through changes in CBF independent of local neuronal activity (Hamel, 2006; Ozbay et al., 2019). Thus, the fMRI changes associated with autonomic activity and arousal changes may be widespread and should be interpreted caution (Soon et al., 2021; Gu et al., 2022). In this regard, joint recording of various physiological signals is recommended, including respiratory rate, depth and inspiratory flow rate using a chest/abdominal belt, heart rate and peripheral vascular tone using photoplethysmography (Allen, 2007), sudomotor activity as measured by skin conductivity (Critchley, 2002) and pupil diameter, eyelid and eye movement using a video camera (Mathot, 2018) (see Figures 2, 3). These signals differently represent various types of autonomic activity and neuro-modulatory tone of the cortex, and their joint analysis may help separate their effect on the fMRI signal. In this regard, it may be particularly useful to combine fMRI with electro-encephalography EEG, which helps identifying the neuro-electrical contributions to fMRI signal changes with autonomic activity (Picchioni et al., 2022).

Finally, another aspect of the interaction between changes in arousal and autonomic activity that merits consideration is a potential dependence of autonomic regulation on arousal state. For example, baroreflex sensitivity is increased during low arousal states of deep sleep, while operating at a lower setpoint, i.e., requiring larger blood pressure drops to engage (de Zambotti et al., 2018).

During rapid eye movement (REM) sleep, coupling exists between PGO waves and cardiac and respiratory activity (Snyder et al., 1964; Orem, 1980b). During non-REM sleep, coupling is observed between K-complexes, spindles and autonomic activity (Lecci et al., 2017). Arousal state should therefore be considered when performing and interpreting fMRI studies of the CAN. Conversely, fMRI may allow the characterization of variations in AR across different arousal states.

### 3.4 CAN activity, arousal, and learning

AR in part serves to generate a (sometimes anticipatory, see (Sterling, 2012)) adjustment of physiological state to environmental or mental challenges. In the fMRI literature, this adjustment has been associated with a transitioning between distinct brain activity patterns (Raut et al., 2020; Gu et al., 2021; Raut et al., 2025) representing distinct brain states: a default mode (or interoceptive) versus an action mode (or exteroceptive) of brain function (Dosenbach et al., 2025). Such switches have been found to be accompanied by changes in both autonomic and cortical arousal (Liu et al., 2018; Raut et al., 2020; Gu et al., 2022). Although still very preliminary, several recent pieces of evidence suggest that these joint changes may support memory function. For example, arousal changes (inferred from pupil diameter changes) have been found to align with hippocampal memory activity (ripples and replay events) in mice (Yang et al., 2025). Recent research on sensory information encoding and memory retrieval in awake humans also indicate connection with pupil diameter (Yang et al., 2026). Further evidence a comes from low arousal states and sleep (Chen et al., 2022; Bolt et al., 2025). For example, electro-encephalographic (EEG) features such as spindles and K-complexes have been tied to joint cortical and autonomic arousal changes and memory consolidation (Naji et al., 2019; Colrain, 2005; Lecci et al., 2017; Picchioni et al., 2022), potentially facilitated by fluctuations in adrenergic and/or cholinergic neuromodulatory activity (Lecci et al., 2017; Hasselmo, 1999). Similarly pontine-geniculate-occipital waves during REM sleep have been linked to memory functions (Rowe et al., 1999; Ramirez-Villegas et al., 2021; Shuster et al., 2025) as well as heart rate surges (Rowe et al., 1999) and increased respiratory neuron activity (Orem, 1980a). Retrograde tracing shows connections from the sympathetic ganglia to the hippocampus (Westerhaus and Loewy, 2001) indicating a potential influence of autonomic activity on hippocampal function. Combined these studies suggest an association between autonomic activity and memory function that is reflected in the fMRI signal.

### 3.5 Autonomic activity and brain fluid homeostasis

Brain fluid homeostasis refers to the process of regulating the distribution and concentration of solutes in the brain’s cerebrospinal fluid (CSF) and interstitial fluid to support healthy brain function. Among the major homeostatic roles are the supply of nutrients and the removal of waste products through the vasculature (including the choroid plexus) and meningeal lymphatic vessels. An active area of recent research has started to reveal how cerebrovascular pulsations and tone changes may aid brain fluid homeostasis, with potential relevance for revealing the relationship between homeostatic deficiencies and neurological disorders as well as for optimizing therapeutic drug delivery to the CNS (Kipnis et al., 2025). CSF movement induced by the cerebrovascular pulsations and tone changes is thought to aid brain fluid homeostasis by enhancing exchange between CSF and interstitial fluid and mixing with CSF compartments (Kipnis et al., 2025). Major sources for vascular pulsatility include cardiac and respiratory cycles, whereas the slower tone changes originate primarily form neurogenic and non-neurogenic effects on vascular smooth muscle cells (Hamel, 2006). Autonomic activity can affect all these sources of CSF movement, for example, by changing rate and amplitude of cerebrovascular pulsations, or by changing vascular tone through neuro-vascular, chemo-vascular (e.g., vasodilatory effects of CO_2_), or sympathetic mechanisms vascular (Hamel, 2006).

MRI can play important role in understanding of the relationship between CAN activity, autonomic activity and arousal, and CSF movement. For example, fMRI studies can be sensitized to flow of ventricular CSF through the so-called “inflow effect”, allowing the study of the dependence of CSF flow on changes in vascular tone. This is based on the use of fMRI signal amplitude in parenchymal tissue as a proxy for vascular tone (Fultz et al., 2019; Picchioni et al., 2022). These studies underscore the notion that CAN activity can affect CSF flow through changes in vascular tone, including purely chemogenic changes (Picchioni et al., 2022). In addition, MRI can be sensitized to subtle CSF and brain tissue movement through so called “velocity-encoding” (Poncelet et al., 1992; Wang et al., 2022). Comparison of the various types of pulsations shows that those origination from the cardiac cycle lead to the smallest CSF displacements but are most frequent, whereas those originating from CO_2_ mediated changes in vascular tone are largest but slow and infrequent (Wang et al., 2022).

## 4 Summary and outlook

Advances in high field MRI have helped improve resolution and further improvements are expected with ongoing developments in gradient hardware. Recent studies have demonstrated the ability to explore anatomical and functional connectivity within and with the CAN and have confirmed previous knowledge from invasive studies. Increasingly, fMRI studies are revealing the complex and context-dependent set of brain regions that coactivate with autonomic changes. Interpretation of these coactivation patterns therefore requires consideration of this context and is helped by the collection of multiple physiological indicators such as respiratory flow, cardiac rate, and peripheral vascular tone. In studies with clinical focus, additional indicators may be valuable as well: for example, a cutaneous electro-gastrogram or MRI measures of gut movement can be used in the study of gut-brain interactions and pathological function in, e.g., functional dyspepsia (Rebollo et al., 2018; Sclocco et al., 2022). Interpretation of fMRI studies of autonomic activity should also consider the high variability of autonomic physiology even in healthy subjects. This variability in part relates to the multiple interacting pathways that ensure redundant control of the autonomic mechanisms that control homeostasis. The relative activity of these pathways may be affected by various factors, including arousal state, psychological state and physiological baseline. In addition, the generative mechanisms underlying fMRI contrast may be affected by drugs such as caffeine and betablockers and other vasoactive agents. Lastly, the fMRI environment itself should be factored into this interpretation: body pose does affect autoregulatory parameters.

Intriguing recent MRI research has shown an association between autonomic activity, arousal, and learning. While this association complicates efforts aimed at mapping the CAN, it may help understand how the brain may switch between operational modes to optimally balance (environ)mental demands and homeostatic functions. Unfortunately, temporal resolution limits inherent to fMRI contrast generally prevent making causal inferences about the role of the various processes and brain activities covarying with autonomic activity. Possibly, invasive studies in animals will shed a light on this aspect. Ultimately, improved understanding the interactions within CAN and between CAN other brain regions may help understand the various pathological processes underlying neurodegenerative disease (Mather, 2025).
